# Dr. J. McF. Gaston

**Published:** 1884-03

**Authors:** 


### PERSONAL.

DR. J. MCF. GASTON, whose excellent and instructive papers, from Brazil, gave so much pleasure, has returned to his "country" again; not to the Palmetto State, but to the one adjoining. His address is now 38½ Broad street, Atlanta, Ga. It is very probable that he will give to the American public, through this JOURNAL, his reminiscences of Brazil, and take the opportunity, while so doing, of presenting much of the valuable results of his varied experience in his recent home.—*Gaillard's Medical Journal.*

We are pleased to announce that the Doctor will contribute an occasional article to the ATLANTA MEDICAL AND SURGICAL JOURNAL.

